# The Development of Selective Attention in Children's Emotion Reasoning

**DOI:** 10.1111/desc.70167

**Published:** 2026-03-11

**Authors:** Andrea G. Stein, Seth D. Pollak

**Affiliations:** ^1^ Department of Psychology University of Wisconsin‐Madison Madison Wisconsin USA

**Keywords:** distributed attention, emotion learning, emotion reasoning, middle childhood, selective attention, strategic variability

## Abstract

We investigated developmental differences in the attention strategies children use in their emotion reasoning across middle childhood. Specifically, we examined whether, with age, children become less likely to distribute their attention broadly when reasoning about emotions, and more likely to come to attend selectively to the most reliable cues. To test this hypothesis, *N* = 180 five‐ through ten‐year‐old children learned to classify an alien's emotional displays into two categories. Categories could be learned via two different strategies: distributing attention across all possible emotion‐related cues or selectively attending to the most predictive cue. Consistent with our hypothesis, Bayesian zero‐one inflated beta regression revealed that from five through approximately eight years old, with age, participants were increasingly likely to use a selective attention strategy. Selective attention use increased by an average of 5 percentage points per year across this age range (95% CI [2%pt, 7%pt]). This result was also characterized by decreasing strategic variability across development. For every year older a participant was, they were also 1.77 times more likely to use a single consistent attention strategy, rather than a hybrid of distributed and selective attention (95% CI [1.43, 2.22]). These results reflect a potential trade‐off across development between flexibility and efficiency in emotion reasoning.

**Summary:**

- There are many different cues we can use to reason about others’ emotions; the ways we attend to such cues may vary across development
- We investigated children's attention strategy use during emotion reasoning in the context of a novel emotion category learning paradigm
- From ages five through eight, with age, children were increasingly likely to attend selectively to the most reliable emotion cue, rather than distribute their attention
- This shift may give rise to a changing trade off across middle childhood between flexibility and efficiency in emotion reasoning

## 1 Introduction

Humans are constantly making inferences about what other people might be feeling. These computations are so common and seem so intuitive that we understand relatively little about how these skills emerge. Imagine watching someone working on a challenging task and trying to decide whether or how to intervene. This decision is likely to depend upon how you think that person is feeling. There are many pieces of information that you might consider to make such an inference, such as how well they seem to be doing with the task, whether they are being observed by others, what they are doing with their face and body, what sounds they are making, and how important the task is—to name only a few. Also available are a vast array of details that you might not attend to as relevant such as the color the person is wearing, their hairstyle, the furniture in the room, or (unless relevant to the task) features such as their height or handedness. Emotion reasoning tasks like these are complex because there are always myriad cues that might help us understand how someone else is feeling. How do humans come to determine how to weight different signals when reasoning about emotions? Here, we investigate developmental changes in the ways we approach the multitude of cues available to us when reasoning about others’ emotions.

### 1.1 Developmental Differences in Facial vs. Contextual Cue Use

Most research into the development of cue use during emotion reasoning has focused on developmental differences in how individuals weigh different types of emotion‐related cues. In particular, many studies have focused on the relative weight that children and adults give to facial configurations compared to contextual cues. Research in this area (e.g., Leitzke et al. 2025; Leitzke and Pollak 2016; Mondloch 2012; Mondloch and Horner 2013; Nelson and Mondloch 2017) has generally presented participants with stimuli in which a character's face putatively pointed to one emotion category, while contextual cues—such as body postures or the situational context—pointed to another, and asked them to infer the character's emotion.

These studies generally find that both children and adults take context into account in their emotion reasoning—even when explicitly instructed not to—as long as stimuli involve emotions of the same valence. However, across childhood, this effect of context appears to decline, while faces gain increasing primacy. Furthermore, complementary eye‐tracking evidence suggests that in many emotion reasoning tasks, children tend to spend less time looking to faces than adults, and more time looking to the surrounding context (Leitzke and Pollak 2016; Nelson and Mondloch 2017, 2018, 2019). Taken together, then, this research suggests that with development, facial cues gain primacy.

However, this general approach—examining the relative salience of different types of cues while participants reason about conventional emotion categories—has limitations when it comes to understanding the development of children's emotion reasoning. For one, these studies have relied on prototypical “basic” emotion categories for their investigations. Adults and children may have different understandings of these prototypes and the stimuli used to represent them. However, this potential confound is often insufficiently addressed. For example, studies have not always validated facial stimuli from sets previously normed by adults when testing child participants (e.g., Mondloch 2012). Furthermore, when such stimuli were validated within a given study, in many cases face‐emotion category and context‐emotion category associations were inconsistent among children and liable to vary developmentally (e.g., Leitzke and Pollak 2016). Finally, even when such checks were in place, extant studies have not been able to fully prevent participants’ emotion knowledge from confounding results in more complex ways. For example, while most participants might label both a putatively angry face and a putatively anger‐associated context as anger when norming stimuli, this result does not guarantee these two associations with anger are equally strong (or only unequally strong because of something about the nature of faces vs. contexts in general). By using conventional emotion categories, these studies thus introduced the potential confound of participants’ existing emotion knowledge.

### 1.2 Developmental Differences in Distributed vs. Selective Attention Strategies

Focusing on developmental changes in the relative saliency of different types of cues may also belie more general developmental differences in how children use information from the environment to reason about emotions. In particular, cognitive development research suggests there may also be developmental changes in the broader attention strategies children use when reasoning about emotions (Best et al. 2013; Deng and Sloutsky 2015, 2016). Specifically, across development, children may become increasingly likely to use *selective attention strategies—*strategies in which the individual focuses on the cue(s) from the environment they believe are the most informative for the task at hand, while filtering out other, presumably less informative cues. Correspondingly, they may become less likely to use *distributed attention strategies*—strategies that entail spreading one's focus across a wider range of environmental cues.

For example, Deng and Sloutsky (2016; Study 1) tasked four‐year‐olds, seven‐year‐olds, and adults with classifying two species of aliens. One of the aliens’ features was perfectly predictive of species membership, while all the other features were moderately predictive. Thus participants could learn to classify the aliens according to either a selective attention strategy, in which they relied on only the perfectly predictive feature, or a distributed attention strategy, in which they relied on all of the features. Following training, participants classified ambiguous stimuli in which the value of the alien's perfectly predictive cue pointed to one species, but the majority of cues pointed to the other. From participants’ classification decisions, the researchers were able to assess participants’ attention strategy use. They found that 4‐year‐olds were more likely to use a distributed attention strategy, while 7‐year‐olds and adults were more likely to use a selective attention strategy.

This shift from more distributed to more selective attention across development is not specific to category learning. Similar trends have been observed across a variety of paradigms, including change detection and target detection tasks (Goldberg et al. 2001; Plebanek and Sloutsky 2017, 2019) visual search tasks (Plebanek and Sloutsky 2017), memory interference paradigms (Darby et al. 2021), and negative priming paradigms (Pritchard and Neumann 2011). The specific developmental time‐course of this distributed‐to‐selective shift, however, appears to depend on not only the type of paradigm, but also its specific demands. In higher cognitive load tasks, adult‐like selective attention use may not be observed until adolescence; however, in many other cases, children's patterns of selective attention use approach those of adults in middle childhood (Pritchard and Neumann 2011; Rueda and Posner 2013). Meanwhile, the time course of such a shift in the context of emotion reasoning—if such a shift does in fact take place—remains unknown.

### 1.3 Implications of Distributed vs. Selective Attention Strategies

Research in nonemotional domains also suggests that the attention strategies children use may have important implications for reasoning. This research finds that attention strategy use involves an important trade‐off, not unlike the explore–exploit trade‐off in the reinforcement learning literature (e.g., Gopnik 2020) or the learning‐performance trade‐off in the brain development literature (e.g., Thompson‐Schill et al. 2009). Namely, as we detail here, attention strategy use involves a trade‐off between the flexibility provided by distributed attention and the efficiency provided by selective attention (Blanco et al. 2023; Hoffman and Rehder 2010; Plebanek and Sloutsky 2017).

The major benefit of selective attention is its efficiency; by definition, selective attention requires processing a more limited set of information. Consistent with this benefit, during category learning tasks, adults tend to engage in attention optimization, coming to narrow their attention to the fewest possible cues (Rehder and Hoffman 2005). In fact, computational modeling suggests that adults’ behavior is often best fit by models that over‐regularize—that is, models that reduce the number of cues they incorporate, even at the expense of some degree of accuracy (Galdo et al. 2022). The efficiency provided by selective attention could be very useful for emotion reasoning, given the sheer number of emotion inferences we make in our day‐to‐day lives, and the speed with which we generally must do so.

In contrast, the major drawback of selective attention is its lack of flexibility. Selective attention involves ceasing to attend to many cues from the environment, which can have both immediate and longer‐term consequences (Blanco and Sloutsky 2019; Hoffman and Rehder 2010). In the immediate term, cues that are not attended to are not learned about, even though may be turn out to be useful later. Furthermore, in the longer term, selective attention can lead to learned inattention, wherein once an individual learns to ignore a given cue, they become less able to detect if it subsequently becomes meaningful in the future. Accordingly, selective attention can be particularly risky in highly variable environments—especially environments in which the relative informativeness of different cues is liable to change (Best et al. 2013; Blanco et al. 2023; Plebanek and Sloutsky 2017). It may also be pernicious in environments characterized by complexity—in particular, environments in which accurate reasoning requires relying on more complicated combinations of cues than an individual might initially assume (Rich and Gureckis 2018). Distributed attention, meanwhile, gives the individual more flexibility to learn about a broader range of cues, notice changes in their environment, and adjust their beliefs about the informativeness of different cues with new information. For these reasons, attention strategy use may have profound implications for the development of emotion reasoning.

### 1.4 The Current Study

Given the complexity and variability of learning environments for emotion (see, e.g., Barrett et al. 2019; Ruba et al. 2022), the flexibility provided by distributed attention could be hugely beneficial for emotional development. However, research has yet to determine whether the development of children's emotion reasoning is characterized by the same shift from distributed to selective attention strategy use found in other domains. We reasoned that the flexibility provided by distributed attention could be critical for younger children, who, because of their comparative lack of experience, likely have less knowledge about individual emotion cues, less well‐developed theories of how emotions work (i.e., which cues might be useful in which situations), and less of a sense of the stability vs. variability of their emotional environment. Accordingly, we hypothesized that selective attention use during emotion reasoning would increase with age in middle childhood.

To test this hypothesis, we adapted Deng and Sloutsky's (2016) category learning paradigm into a novel emotion category learning task. We tested whether younger children are more likely to distribute their attention widely across a variety of cues as they reason about emotions, while older children are more likely to come to attend selectively to the most predictive cues. Such a result would support possibility that the shift from distributed to selective attention observed in other domains also takes place in the children's emotion reasoning development. For our initial study of this question, we tested five‐ through ten‐year‐olds, a range that maps onto previous research on the distributed‐to‐selective attention shift in nonemotional domains. If our hypothesis was supported by the data, it would suggest that children's emotion reasoning development entails change in not only what kinds of cues children rely on most, but also in how narrowly vs. broadly they consider the many cues they encounter.

## 2 Method

### 2.1 Participants

Participants were five‐ through ten‐year‐old children (*M*_age_ = 7.99 years, *SD*_age_ = 1.73 years) recruited on the online developmental research platform Lookit/Children Helping Science (Scott and Schulz 2017) between April and October 2024. Lacking prior data to meaningfully inform simulations for a Bayesian approach to sample size planning, and lacking guidance specific to zero‐one inflated beta regression models, we determined our sample size based on Smithson and Verkuilen (2006), who suggest that using sample sizes of over one hundred in the context of standard beta regression models may minimize bias. Accordingly, our sample consisted of *N* = 180 participants (30 per one‐year age bin). Of these 180 participants, 53% identified as female, and 47% as male. 55% of participating families identified as White; 17% as Asian; 4% as Hispanic, Latino, or of Spanish origin; 2% as American Indian or Alaska Native; and 19% as multiracial. 3% of families did not report this information. For 8% of participants, their caregivers’ highest level of education was a high school diploma; for 4%, an associate degree; for 34%, a bachelor's degree; and for 49%, an advanced degree. 5% of families did not report this information. Data from an additional 85 children were collected but not included in analyses following review of participants’ video recordings for data quality control. 32 were excluded due to response interference by family members, 24 were excluded due to excessive distractions in their home environment during the task, 18 were excluded due to technical issues (e.g., problems with their video recordings), and 11 were excluded due to incomplete data.

All participating families provided video‐recorded informed consent, and all participating children provided screen‐captured assent. All participants received a $10 Amazon.com gift card. This study was approved by the University of Wisconsin‐Madison Institutional Review Board.

### 2.2 Materials

Stimuli were computer‐graphic designed images of an alien (“Wiggle the Wuggle”) made up of five binary features on their face and in the surrounding context: Wiggle's eyes (narrowed vs.widened), Wiggle's mouth (smiling vs. pursed), the setting (at home vs. at school), the social context (with a child vs. with an adult), and the activity (playing with a ball vs. reading a book). To minimize the potential confounding effect of participants’ prior knowledge about human emotions, facial features were designed to avoid combinations of facial action units prototypically associated with human emotions (see Barrett et al. 2019). A validation survey among adults using an earlier version of these facial features (featuring a slightly narrower mouth for the category 1 prototype) confirmed that individuals did not consistently map these features to specific emotion categories. Furthermore, to the extent that they did so, these mappings were similar across categories, rather than serving to distinguish them. For both combinations of facial features, the most commonly provided label was “happy,” but this label was provided by only 20% of participants for the first combination, and 33% of participants for the second. Meanwhile, contextual features for the stimuli (i.e., setting, social context, and activity) were designed to be low‐information, but plausibly emotion‐related. We chose to compose our stimuli out of five features because this was the lowest possible number of features that would still allow us to develop multiple ambiguous stimuli in which one cue (the perfectly predictive cue) pointed to one category, while the majority of cues still pointed to the other. Piloting showed that adding features beyond these five made it substantially more difficult for young participants to learn the categories—a prerequisite for our main analyses.

**FIGURE 1:**
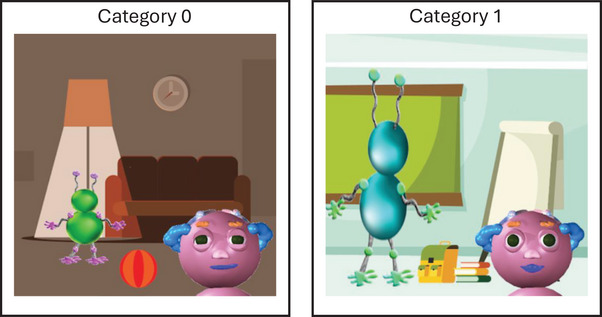
Emotion category prototypes *Note*: Emotion category prototypes varied in terms of five features on Wiggle's face and in the surrounding context: Wiggle's eyes (narrowed vs. widened), Wiggle's mouth (smiling vs. pursed), the setting (at home vs. at school), the social context (with a child vs. with an adult), and the activity (playing with a ball vs. reading a book).

**FIGURE 2:**
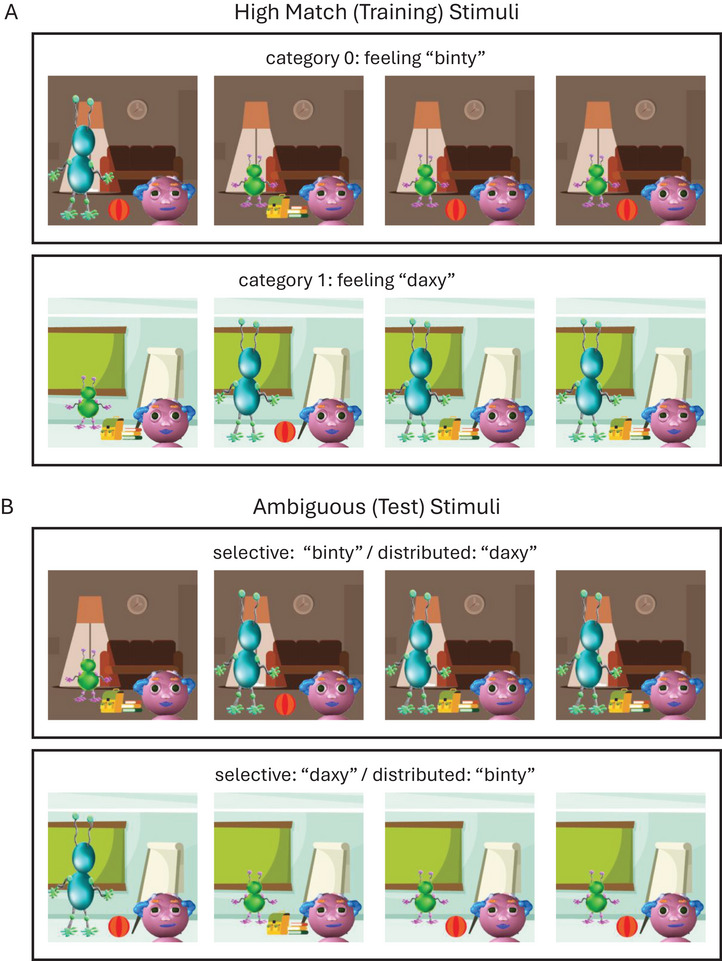
Example stimulus set *Note*: (A) High match stimuli used during training. In this example, category 0 was randomly assigned to the emotion label “binty”, and category 1 to the emotion label “daxy.” Likewise, setting was randomly assigned as the perfectly predictive cue, matching the corresponding category prototype in every stimulus in the training set. The other four cues were moderately predictive of category membership, matching the corresponding category prototype in 75% of stimuli in the training set. (B) Ambiguous stimuli shown during the test phase to assess participants’ attention strategy use. For each stimulus, the perfectly predictive cue (in this example, setting), corresponded to one category prototype, but the majority of cues corresponded to the other. For stimuli in the upper panel, the perfectly predictive cue (setting) matched the category 0 prototype (and the emotion label “binty”), but the majority of cues matched the category 1 prototype (and the emotion label “daxy”). Thus a response of “binty” would suggest selective attention use, and a response of “daxy” would suggest distributed attention use. For stimuli in the lower panel, these relations were reversed.

These five features were used to create two category prototypes—one in which each feature took on its first possible value, and one in which each feature took the other value (see Figure 1). Using these prototypes, we created five different stimulus sets, so any one of the five features could be randomly assigned as the perfectly predictive cue. In each stimulus set, there were eight “high match” stimuli (four per category) in which the perfectly predictive cue always matched the category prototype, and three of the other four (“moderately predictive”) cues also matched that prototype. These stimuli were presented during the training phase to allow participants to learn the categories. There were also eight “ambiguous” stimuli, in which the perfectly predictive cue matched one category prototype, but three of the other four moderately predictive cues (and thus also the majority of the five cues) matched the other. These stimuli were presented during the test phase to assess whether participants used a selective attention strategy or a distributed attention strategy in the task. See Figure 2 for an example stimulus set.

### 2.3 Procedure

#### 2.3.1 Task Procedure

We used stratified random sampling within each one‐year age bin to assign (a) which of the five features was the perfectly predictive cue; and (b) which category prototype corresponded to the emotion label “binty,” and which corresponded to the emotion label “daxy”. All participants completed the study online and were video recorded; these videos were later reviewed by researchers for data quality control.

After consent and assent, participants were introduced to Wiggle the Wuggle. Children were told that, like humans, Wuggles have feelings, but these feelings look different from and have different names than human feelings (specifically, “binty” and “daxy”). Children were next introduced to the task and oriented to the five features they might use to make their categorization decisions. By design, we did not identify which feature was the perfectly predictive cue. Following each instructions block, participants completed a comprehension check.

Participants completed 72 task trials (eight blocks of eight training trials, and one block of eight test trials). In each training block, they were presented with the eight high match stimuli from their stimulus set in a random order. On each trial, they were asked to indicate whether Wiggle was feeling binty or daxy in the corresponding picture, then received corrective feedback. The test block was identical to the training blocks, except that instead of being presented with the eight high match stimuli, participants were presented with the eight ambiguous stimuli from their stimulus set in a random order. Because there was no canonically correct answer for these stimuli, but piloting showed that ceasing to provide feedback led younger participants to become discouraged and disengaged, feedback during the test block was always positive. Results of post hoc logistic regression showed that there was no credible increase or decrease in participants’ selective attention strategy use over the course of these eight ambiguous trials, suggesting this positive feedback did not bias participants toward use of a particular attention strategy. Blocks were not differentiated for participants, except that breaks were built in after the third and sixth training blocks.

#### 2.3.2 Video Review Procedure

Video recordings were reviewed by trained research assistants to note instances of: (a) interference with the participant's responses (e.g., a family member gave the participant a hint, or weighed in on which answer they should choose); (b) excessive distractions in the environment (e.g., a family member held an unrelated conversation with the participant during the task, there were excessive disturbances in the background, or the participant was distracted by another activity while completing the task); (c) technical issues (i.e., the participant had a technical issue with the study or, more commonly, there was a technical issue playing back their video); and (d) incomplete data (i.e., the participant did not finish the task). The first author reviewed videos with these indicators to make final determinations regarding exclusions. At the time of review, research assistants and the first author were naïve to participants’ strategy use.

### 2.4 Analysis Plan

Our analyses consisted of (a) preliminary analyses of participants’ category learning in the final block of training, confirming that learning took place; (b) main analyses of participants’ responses to ambiguous stimuli post‐training, testing our hypothesis that selective attention use during emotion reasoning would increase with age; and (c) replication of our main analyses in the subsample of participants who exhibited high category learning. Analysis scripts and deidentified data are available on OSF: https://osf.io/mre8n/. Analyses were conducted in R version 4.5.0.

For each set of analyses, we used inflated beta regression modeling. Beta regression models are particularly well‐suited for modeling proportion outcome variables (such as the proportion of correct responses at the end of training, or the proportion of responses at test consistent with a particular attention strategy) because they obviate issues of heteroskedasticity, skew, and out‐of‐bounds estimates associated with linear modeling of proportions data, and provide greater flexibility than fractional logistic regression of proportions data with regard to the shape of the outcome distribution (Ferrari and Cribari‐Neto 2004; Smithson and Verkuilen 2006). Zero‐one inflated beta regression models, in contrast to standard beta regression models, also allow for estimation of outcome proportions that are exactly 0 and exactly 1 (i.e., 0% or 100% accuracy; 0% or 100% selective attention use). Furthermore, because zero‐one inflated beta regression models are mixture models of four different sub‐models, they allow the generative processes for these {0,1} values to be distinct from the generative process for values on the (0,1) interval. This parametrization entails that, in our main analyses, the factors leading someone to use a completely consistent selective attention strategy or a completely consistent distributed strategy could be distinct from the factors determining the relative proportions of selective vs. distributed attention strategy use for those using a mix of the two strategies.

For all analyses, we used Bayesian methods, as implemented in the R package brms (Bürkner 2017), to fit the models. All models were estimated using brms's default priors. Models were run for four MCMC chains of 2000 iterations each, the first 1000 of which were discarded as warm‐up. For each model, we checked for convergence via visual inspection of trace plots and autocorrelation plots, as well as estimation of potential scale reduction factors and effective sample size ratios. We also performed posterior predictive checks for the mean, the proportion of 1 outcomes, and, in the case of our main analyses, the proportion of 0 outcomes, as well as visually inspecting posterior predictive distributions.

Based on the previously reviewed literature, we expected it was possible that task behavior would differ depending on whether a participant had a contextual cue (activity, setting, or social context) or a facial cue (eyes or mouth) as their perfectly predictive cue. We also expected that such an effect of perfectly predictive cue condition on task behavior might itself vary developmentally. Thus, for both sets of analyses, we started by performing Bayesian model comparison to select the model with best predictive performance out of nested zero‐one inflated beta regression models involving participant age, perfectly predictive cue condition, and their interaction. In all of these models, perfectly predictive cue condition was coded (−0.5, 0.5), with −0.5 corresponding to those who had a contextual cue as their perfectly predictive cue, and 0.5 corresponding to those who had a facial cue as their perfectly predictive cue. In all models, participant age was mean‐centered. For each set of analyses, we report the results of the model that performed best in terms out‐of‐sample predictive performance (i.e., the model with the lowest leave‐one‐out cross‐validation information criterion [LOOIC]).

To support the appropriateness of our use of the zero‐one inflated beta regression generative structure empirically, we also performed the same main analyses reported here but instead used standard beta regression after bringing all 0 and 1 outcomes to near‐0 and near‐1 values using a transformation suggested by Smithson and Verkuilen (2006). Unlike the models reported here, these standard beta regression models consistently failed posterior predictive checks because they underestimated the proportions of both near‐consistent distributed attention use and near‐consistent selective attention use. Full results of both sets of model comparisons are available on OSF: https://osf.io/mre8n/.

For each set of analyses, to aggregate effects across sub‐models, we estimated model predictions and marginal effects of model parameters using the R package marginaleffects (Arel‐Bundock et al. 2024). When interpreting these effects, we considered all effects whose 95% equal‐tailed credible intervals excluded zero to be credibly non‐zero. We used equal‐tailed credible intervals, rather than highest density intervals, to enable transformations between log‐odds and odds for some parameter estimates.

## 3 Results

### 3.1 Preliminary Analyses: Category Learning Performance

First, we sought to confirm that participants exhibited sufficient category learning to make our main analyses of their attention strategy use meaningful. Among the models we compared predicting participants’ accuracy on the final training block, the Bayesian one‐inflated beta regression model including age and perfectly predictive cue condition (but not their interaction) performed best. The model did not include a zero‐inflated component, because no participants achieved 0% accuracy. This model passed all convergence checks (all *r̂* < 1.1; all *N_eff_/N* > 0.1) and posterior predictive checks (all posterior predictive *p*‐values: 0.40 < *p* < 0.60).

We then used this model to estimate the predicted performance of the average participant (i.e., the combined intercept, representing the predicted performance of a participant of mean age and hypothetically in between perfectly predictive cue conditions). The average participant was predicted to have 83% accuracy in the final training block (95% CI [79%, 87%]). Thus, while not consistently performing at ceiling by the end of training, participants’ performance was credibly well above chance (50%), suggesting category learning did take place.

To characterize any individual differences in category learning performance, we also estimated the average marginal effects of age and perfectly predictive cue condition. Category learning improved with age. On average, for every year older a participant was, they were predicted to be 3 percentage points more accurate in the final training block (95% CI [2%pt, 5%pt]). Category learning also appeared to be easier in the facial perfectly predictive cue condition than in the contextual condition. On average, participants who had a facial cue as their perfectly predictive cue were predicted to be 18 percentage points more accurate in the final training block than those who had a contextual cue as their perfectly predictive cue (95% CI [12%pt, 23%pt]).

### 3.2 Main Analyses: Developmental Differences in Attention Strategy Use

Next, we sought to test our central hypothesis that selective attention use would increase with age. Among the models we compared predicting the proportion of participants’ responses to ambiguous stimuli at test consistent with selective attention strategy use, the Bayesian zero‐one inflated beta regression model including age and perfectly predictive cue condition (but not their interaction) performed best. This model passed all convergence checks (all *r̂* < 1.1; all *N_eff_/N* > 0.1) and posterior predictive checks (all posterior predictive *p*‐values: 0.40 < *p* < 0.60).

**FIGURE 3:**
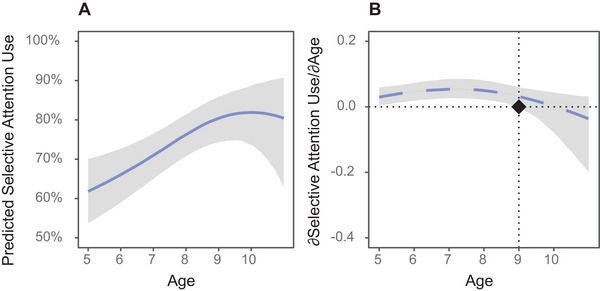
Predicted selective attention use and marginal effects of age on selective attention use, by age *Note*: Estimates correspond to the median of the corresponding posterior distribution. Error regions represent 95% equal‐tailed credible intervals. All analyses control for perfectly predictive cue condition at its center. Panel (A): Predicted selective attention use by age. Panel (B): Rate of change in selective attention use by age (i.e., marginal effect of age, by age). Values above zero correspond to increasing selective attention use with age; values below zero correspond to decreasing selective attention use with age. The black diamond indicates the age at which the corresponding 95% credible interval no longer exceeds zero—that is, the age at which there is no longer a greater than 95% posterior probability that selective attention use is increasing with age.

We then estimated model predictions of selective attention use by age, controlling for the effect of perfectly predictive cue condition at its center (see Figure 3(A)). Overall, the model predicted high proportions of selective attention use, ranging from 62% selective attention use at age 5;0 (95% CI [54%, 70%]), to 82% selective attention use at its peak at age 9;11 (95% CI [74%, 88%]). We then estimated the marginal effects of age on these predictions at one‐month intervals across our age range (see Figure 3(B)). Mathematically, these marginal effects of age corresponded to the partial derivative of the model predictions curve with respect to age, or the rate of change in selective attention use with age. From ages 5;0 through age 8;11, 95% credible intervals for the marginal effect of age excluded and exceeded zero, meaning we can conclude with a high degree of confidence (corresponding to a ≥95% probability) that selective attention use increased with age. Across this age range, participants were predicted to increase their selective attention use by an average of 5 percentage points for every year older they were (95% CI [2%pt, 7%pt]).

From ages 9;0 to 10;11, credible intervals for the marginal effect of age overlapped zero, meaning we cannot conclude with high confidence that selective attention use either increased or decreased with age. To further characterize this result, we sampled from the posterior distributions for the marginal effects of age along this range. This sampling revealed a relatively high posterior probability (96%) that selective attention use was still increasing with age at 9;0, a‐near chance posterior probability (51%) that selective attention use was still increasing with age by 10;0; and a low posterior probability (25%) that it was increasing with age at age 10;11.

To provide a more detailed picture of the processes that might underlie these age‐related differences in attention strategy use, we also examined the parameter estimates for age in each of the four sub‐models—namely:

- the *𝛼* sub‐model, which estimated the odds that a participant's responses were characterized by either consistent use of a selective attention strategy or consistent use of a distributed attention strategy, rather than any mix of the two.
- the *𝛾* sub‐model, which estimated the odds that if a participant used a single consistent strategy, that strategy was a selective attention strategy, rather than a distributed attention strategy.
- the *𝜇* sub‐model, which estimated the mean proportion of selective attention use among participants who used a mix of selective and distributed attention strategies.
- the *Φ* sub‐model, which estimated the extent of (in)variance in proportions of selective attention use among participants who used a mix of selective and distributed attention strategies.

Results suggested that increasing strategic consistency across development underlay the observed age‐related differences in attention strategy use. Namely, age had a credible effect on *𝛼*, such that with age, children were more likely to consistently use a single attention strategy (i.e., either selective or distributed attention) for all trials, rather than a mix of the two strategies. For every one year older a child was, they were 1.77 times more likely to use a single strategy consistently (95% CI [1.43, 2.22]). Age did not have a credible effect on *𝛾, 𝜇*, or *Φ*.

### 3.3 Follow‐Up Sensitivity Analyses: Attention Strategy Use in High Learning Subsample

Category learning performance in this sample was high, but not at ceiling. Because category learning improved with age, we wanted to rule out the possibility that the results we observed were partly attributable to response variability as younger children tried to learn the categories (rather than variability in attention strategy use per se). To do so, we reran our primary analyses including only the subsample of participants who achieved at least 85% accuracy in category learning in the final training block (*n* = 104 participants).

Results mirrored those in the full sample. Namely, marginal effect estimates showed credible increases in selective attention use with age from ages 5;0 through 8;0. Across this age range, participants were predicted to increase their selective attention use by an average of 6 percentage points for every year older they were (95% CI [1%pt, 12%pt]). These developmental differences were again characterized by increasing strategic consistency with age: For every one year older a child was, they were 1.68 times more likely to use a single attention strategy consistently, rather than a mix of the two (95% CI [1.22, 2.36]). Full results from these analyses are available on OSF: https://osf.io/mre8n/.

## 4 Discussion

Consistent with our hypothesis, we observed developmental differences in attention strategy use during emotion reasoning such that, from ages five through approximately eight years old, participants were increasingly likely to use a selective attention strategy, rather than a distributed attention strategy, to reason about emotions. This result is consistent with previous findings of a developmental shift from distributed to selective attention use in middle childhood in nonemotional contexts (e.g., Deng and Sloutsky 2015, 2016), and provides evidence that such a shift may represent a domain‐general childhood phenomenon.

This result may also have important implications for our understanding of the development of children's emotion reasoning. Extrapolating from the literature reviewed earlier regarding the costs and benefits of selective attention (e.g., Blanco et al. 2023; Hoffman and Rehder 2010; Plebanek and Sloutsky 2017), this result suggests that across the early part of middle childhood, children may become more efficient in their reasoning about emotion as they increasingly narrow in on the cues that they believe will most help them make accurate inferences about others’ emotions. However, this efficiency may come at a cost to their flexibility. Older children using more selective attention in their emotion reasoning may not take in as much new information about emotion cues as younger children, and they may not be as open to noticing changes to their emotional environment or ways in which their ideas about emotion may be inaccurate or incomplete. These ideas are empirically tractable; future work should investigate whether, as children grow older, and appear to increasingly rely on selective attention during emotion reasoning, they incur such costs.

In this study, we were also able to characterize the processes underlying these developmental differences in attention strategy use during emotion reasoning. Our primary finding was one of increasing strategic consistently (or decreasing strategic variability) across development. That is, younger children were more likely than older children to alternate between using a distributed attention strategy and a selective attention strategy when reasoning about emotions. Furthermore, this result was preserved among the subset of children who exhibited the strongest emotion category learning, suggesting that it could not be attributed to younger children exhibiting response‐level variability from ongoing attempts at category learning. Instead, this result seems to have reflected real switching between the attention strategies themselves among younger children.

While most analyses of distributed vs. selective attention use in category learning tasks have focused on group‐level differences, this finding of high strategic variability among younger children is consistent with subject‐level analyses of a similar category learning paradigm from Blanco and Sloutsky (2019). They found that, at the end of their task, between 38% and 48% of four‐year‐olds were using a mix of distributed and selective attention strategies (defined as between 30% and 70% selective attention use; see also Weichart et al. 2024).

According to traditional accounts (Siegler 2000, 2007), this sort of strategic variability may be indicative of a time of transition, as a new strategy (e.g., selective attention) becomes available, and the individual tries out this new strategy and compares it to existing strategies (e.g., distributed attention). In the present case, our younger participants’ strategic variability may have reflected reaching a point in development in which they were somewhat newly capable of deploying more selective attention when reasoning about others’ emotions, and they were thus still experimenting with being more selective about the cues they considered. From this perspective, then, early middle childhood may be a time when children are exploring not only different cues to emotion, but also different overarching strategies for thinking about emotions. This possibility should be explored with more research into intra‐individual variability in attention strategy use during emotion reasoning, particularly across a younger age range.

These findings should be considered in light of some limitations. For one, the present study tasked participants with learning about emotion categories in which one cue was perfectly predictive of the corresponding emotion. In the real world, no such perfect one‐to‐one relationships between emotion cues and emotion categories exist. In this way, the present study under‐estimates the inherent variability of children's emotion learning environments. Nonetheless, the present study does aim to capture a broader principle of children's learning environments: While no cue to emotion may be perfectly reliable, some cues may be more reliable than others—and children may vary in the extent to which they narrow vs. broaden their focus across such cues. Similarly, the lab‐based task used here provided participants with one, very explicitly delineated emotion reasoning scenario. Future studies should explore how reasoning in such tasks relates to reasoning in similar real‐life scenarios. A final limitation comes from the composition of our sample. In theory, online research should provide new opportunities to reach more representative samples of families without ties to centers of research. In practice, we have clearly not yet realized these opportunities, as White and Asian and highly educated families were more represented in the online recruitment pool. Accordingly, the generalizability of the present results should be considered with caution.

The present study provides new evidence regarding changes taking place in children's emotion reasoning in early middle childhood. Specifically, across this developmental period, there may be changes in not only kinds of cues that children rely on to reason about emotions, but also in the ways in which they use such cues in their reasoning. From ages five through around eight years old, children appear to shift from distributing their attention broadly across different cues to reason others’ emotions, to attending more selectively to only the most predictive cues. Furthermore, this developmental shift may be underlaid by changes in the consistency of children's attention strategy use across middle childhood, as younger children appear more likely to alternate between distributed and selective attention strategies, while older children are more likely to be consistent in their strategy use. These developmental differences may have key implications for children's flexibility and efficiency as emotion reasoners.

## Disclosure

Early analyses of these data were presented at the 2025 Society for Research in Child Development Biennial Meeting.

## Conflicts of Interest

The authors declare no conflicts of interest to disclose.

## Data Availability

The data that support the findings of this study are openly available on OSF at https://osf.io/mre8n/.
